# Direct Immunofluorescence Using Non-Lesional Buccal Mucosa in Mucous Membrane Pemphigoid

**DOI:** 10.3389/fmed.2018.00020

**Published:** 2018-02-08

**Authors:** Mayumi Kamaguchi, Hiroaki Iwata, Inkin Ujiie, Hideyuki Ujiie, Jun Sato, Yoshimasa Kitagawa, Hiroshi Shimizu

**Affiliations:** ^1^Department of Dermatology, Hokkaido University Graduate School of Medicine, Sapporo, Japan; ^2^Department of Oral Diagnosis and Medicine, Hokkaido University Graduate School of Dental Medicine, Sapporo, Japan

**Keywords:** autoimmune disease, direct immunofluorescence, mucous membrane pemphigoid, oral mucosa, autoantibody

## Abstract

Mucous membrane pemphigoid (MMP) is a rare organ-specific autoimmune subepithelial blistering disease with predominantly mucosal erosions, most frequently affecting the gingiva. Erosions in the oral cavity usually result in markedly decreased quality of life. The major autoantigens are BP180 and laminin332, which are components of basement membrane proteins in the skin and mucosa. Diagnosis is usually difficult due to histological destruction of the tissue and low autoantibody titers. In this study, we evaluated the diagnostic value of direct immunofluorescence (DIF) using non-lesional buccal mucosa in seven cases of MMP. In all seven patients, gingival lesions were clinically observed, and in one of the seven patients, buccal lesions were also clinically observed. First, we performed DIF to detect tissue-bound autoantibodies and complement. DIF from non-lesional buccal mucosa revealed linear deposits of IgG and C3 at the basement membrane zone in all cases. To detect autoantibodies, indirect immunofluorescence (IIF), BP180-NC16A ELISA and immunoblotting were performed. Surprisingly, circulating autoantibodies were unable to be detected in any of the cases by ELISA, IIF, or immunoblotting. Furthermore, histological separation was observed in one patient. In conclusion, DIF using non-lesional buccal mucosa was found to be superior to histological and serological tests for diagnosing mucous membrane pemphigoid. The procedure is technically easy and has high diagnostic value.

## Introduction

The prevalence and incidence of autoimmune disorders are increasing, with many people suffering from such disorders. Autoimmune subepidermal blistering diseases, e.g., bullous pemphigoid, mucous membrane pemphigoid (MMP) and epidermolysis bullosa acquisita, are organ-specific autoimmune disorders that are characterized by autoantibodies to components of the skin basement membrane zone (BMZ) (1–4). Clinically, MMP shows predominant mucosal involvement, most frequently affecting the oral cavity, followed by the conjunctiva, the nasal cavity, and the esophagus (4). In the oral cavity, the gingiva is most commonly affected (70% of cases), followed by the buccal mucosa (60%), the palate (27%), and the tongue and lips (13%) (5). Histological analysis shows junctional separation at the BMZ (4, 6). In immunofluorescence microscopy, linear deposits of IgG and/or complement, and sometimes IgA at the BMZ, are characteristic (4, 7). Several autoantigens are involved in MMP, including BP180 (also called type XVII collagen), laminin332, integrin α6/β4 and type VII collagen, although BP180 and laminin332 are the major autoantigens (4, 7).

The diagnosis of MMP is confirmed based on the combination of clinical findings, histological analysis, and immunological findings. Immunological tests reveal tissue-bound autoantibodies by direct immunofluorescence and circulating autoantibodies by indirect immunofluorescence (IIF), ELISA, or immunoblotting (8). Circulating autoantibodies are frequently difficult to detect; several studies reported that the autoantibodies are detected in approximately 40% of cases (5, 9). By contrast, autoantibodies are detected in more than 80% of cases in bullous pemphigoid, which is an autoimmune subepidermal blistering disease in which BP180 is targeted (10, 11). This difference tends to be due to the low titers of the autoantibodies in MMP (4). Recently, we reported the usefulness of mucosal substrates to detect autoantibodies in MMP (12). Furthermore, histological study fails to show junctional separation because of tissue destruction in the fragile oral mucosa. For these reasons, it frequently takes time to make diagnose MMP and start treatment.

In cases that are difficult to diagnose, direct immunofluorescence (DIF) using the patient’s tissue is a valuable test for diagnosing MMP. Although histological analyses generally should be performed on the affected lesions, DIF samples can be taken from perilesional areas in autoimmune blistering diseases (13). Therefore, we can get specimens in which the structure is maintained, so that we can evaluate the tissue-bound autoantibodies. We, here, report the usefulness of DIF on non-lesional buccal mucosa for diagnosing MMP.

## Materials and Methods

### Patients

All the patients were referred to the dermatology department or to the oral medicine and diagnosis department of Hokkaido University Hospital. The patients demonstrated multiple erosions around the gingiva. DIF tests were performed on non-lesional buccal mucosa.

Patients were selected according to the following criteria: (1) clinically, MMP was suspected and (2) DIF was performed on non-lesional buccal mucosa.

The diagnostic criteria for MMP are as follows: (1) clinical findings of blisters and/or erosions, (2) linear deposits of IgG and/or C3 at the BMZ by DIF., and/or (3) circulating autoantibodies detected by IIF using normal human skin as a substrate, BP180-NC16A ELISA or immunoblotting using normal human epidermal extract.

### Hematoxylin and Eosin (H&E) Staining

Hematoxylin and eosin staining was performed using paraffin embedded sections. After the sections were deparaffinized, specimens were stained with Mayer’s hematoxylin (Muto, Tokyo, Japan) for 3 min. After being rinsed in distilled water, the specimens were stained with 1% eosin Y (Wako, Osaka, Japan) for 1 min, followed by dehydration with 99.5% ethanol.

### Direct Immunofluorescence

**Figure 1:**
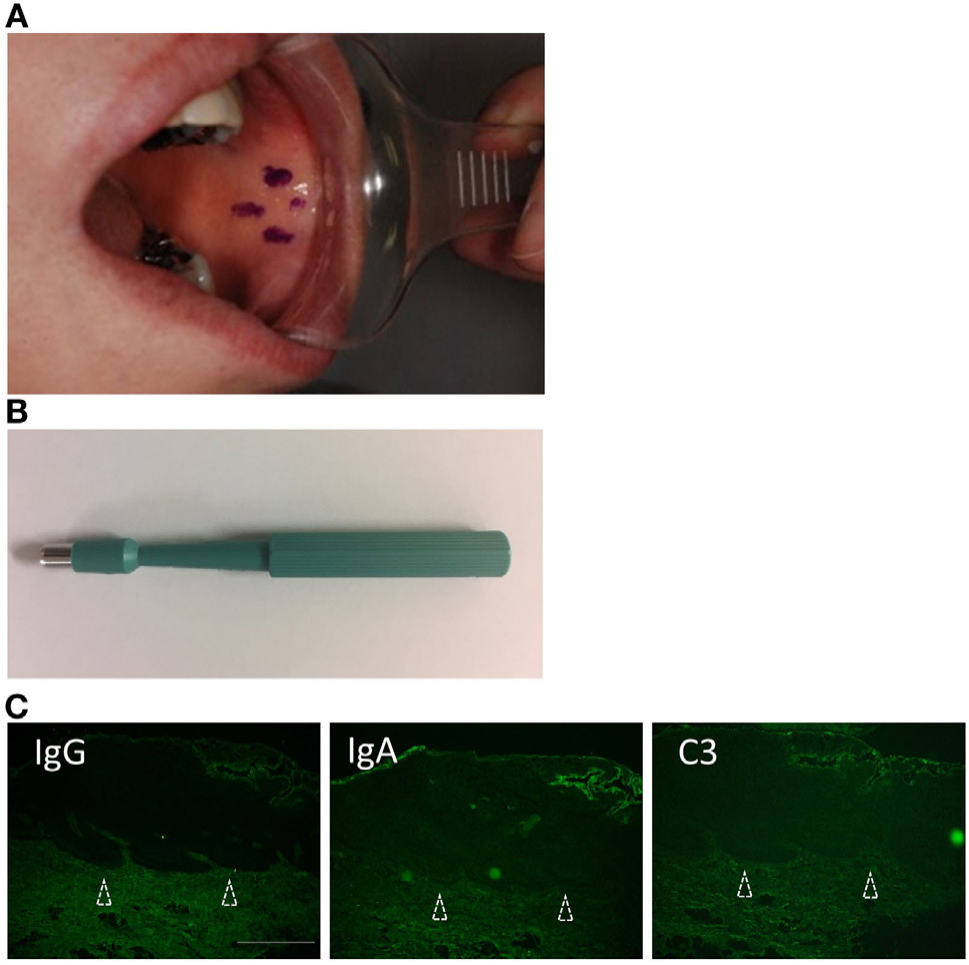
Biopsy from non-lesional buccal mucosa. **(A)** Non-lesional buccal mucosa marked by crystal violet was biopsied under the local anesthesia. **(B)** The samples were taken using a 4-mm punch biopsy tool. No closing sutures were needed. **(C)** A healthy individual shows no evidence of IgG, C3, or IgA. Scale bar = 100 µm.

The specimens were taken from non-lesional buccal mucosa using a 4-mm punch biopsy tool under local anesthesia (Figures 1A,B). The tissues were frozen on the dry ice, and 5-μm-thick sections were prepared by cryostat (Leicabiosystems, Tokyo, Japan). The sections were stained with FITC-conjugated goat anti-human IgG, IgA, IgM, and C3 (1:100, DakoCytomation, Glostrup, Denmark) for 45 min at 37°C.

### Serological Tests to Detect Autoantibodies

Indirect immunofluorescence was performed on normal human skin. The sections were incubated with sera from dilutions of 1:10 to 1:320 for 45 min at 37°C, followed by incubation with 1:100 diluted FITC-conjugated anti-human IgG. Immunoblotting was performed to identify the autoantigens. Normal human epidermal extract was derived as described previously (14). The extracts were applied to 6% SDS-polyacrylamide gel and were then transferred to nitrocellulose membrane. The membrane was blocked for 1 h at room temperature. After incubation with 1:200 diluted sera overnight at 4°C, HRP-conjugated goat anti-human IgG (1:5,000 dilution, Life Technologies, Carlsbad, CA, USA) was reacted for 1 h at room temperature. The BP180-NC16A ELISA was performed using 1:100 diluted sera according to the manufacturer’s instruction (MBL, Nagoya, Japan).

### Statistical Analysis

The sensitivities of the diagnostic procedures were determined including 95% confidence interval (CI). Graph Pad PRISM software Version 7.0 was used to analyze the data.

## Results

### Findings of Tissue-Bound IgG Taken from Normal Buccal Mucosa

**Figure 2:**
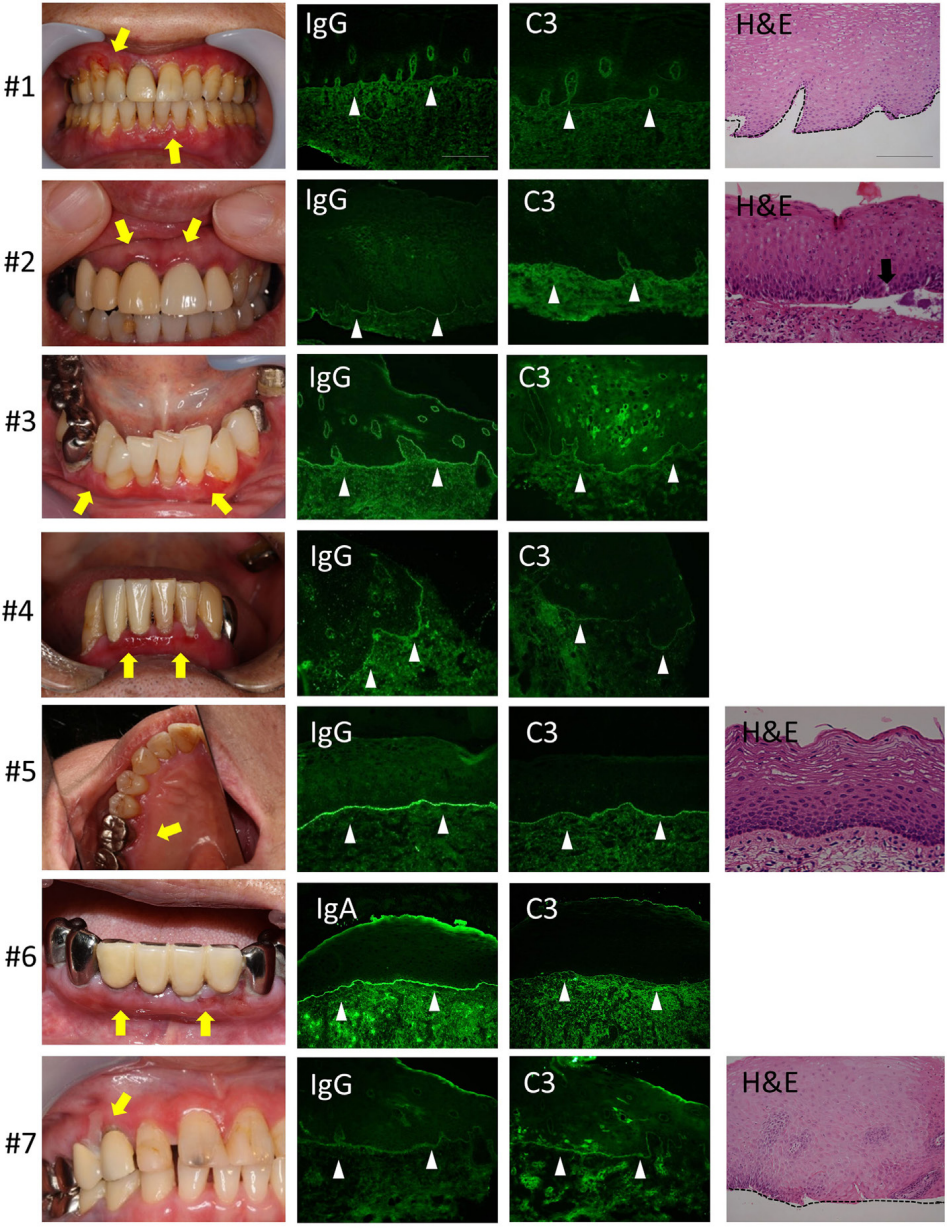
Clinical, histological, and direct immunofluorescence (DIF) findings. All the patients have gingival lesions (yellow arrows). The biopsy samples for DIF were taken from non-lesional buccal mucosa, and all samples shows the linear deposition of IgG or IgA, and C3 at the basement membrane zone (BMZ) in all cases (white arrow heads). Histological analysis shows the junctional separation at the BMZ (#3) (black arrow). The epidermal and dermal tissue are completely separated because of tissue destruction (#1,7). There is no evident separation at the BMZ (#5). Scale bar = 100 µm.

We evaluated seven patients. The clinical findings are shown in Figure 2 and Table 1. All of the patients had gingival lesions, and one patient (case 1) also showed erosions on the buccal mucosa. The mean duration between the initial symptoms and diagnosis was 2.3 years. Four patients received histological analysis, and one patient demonstrated dermal–epidermal separation (#3). In two patients, epidermal and dermal tissues are completely separated because of tissue destruction (#1,7). In the other one patient, there is no evident separation at the BMZ (#5) (sensitivity of H&E: 14.286%, 95% CI: 0.361–56.872). Neither IIF nor BP180-NC16A ELISA was able to detect any circulating autoantibodies (sensitivity of IIF or ELISA: 0%, 95% CI: 0–40.962). We took the DIF samples from the buccal mucosa, and these showed linear deposits of IgG or IgA, and C3 in all cases (sensitivity of DIF: 100%, 95% CI: 59.038–100) (Figure 2). By contrast, the healthy individual showed no evidence of IgG, C3, or IgA (Figure 1C).

**Table 1:** Summary of clinical information.

Case	Age	Disease duration (years)	Skin	Oral cavity	H&E	DIF	IIF	BP180-NC16A ELISA	IB epidermal^a^
1	71–75	3	−	Gingiva, bucal, phalangeal	−	G, C3, A	−	−	−
2	51–55	0.75	−	Gingiva	ND	G, C3	−	−	−
3	66–70	3	−	Gingiva, soft plate	+	G, C3, A	−	−	−
4	76–80	2	−	Gingiva	ND	G, C3	−	−	−
5	71–75	5	−	Gingiva	−	G, C3, A	−	−	−
6	81–85	1	−	Gingiva	ND	G, C3, A	−	−	ND
7	61–65	1.5	−	Gingiva	−	G, C3	−	−	ND

Mean	70	2.3							

*^a^Immunoblotting using normal human epidermal extract*.

*H&E, hematoxylin and eosin stain; DIF, direct immunofluorescence; IIF, indirect immunofluorescence; G, IgG; A, IgA; ND, No data*.

## Discussion

Direct immunofluorescence showing tissue-bound antibodies is the strongest evidence for antibody-induced autoimmune diseases. Such immunofluorescence reveals deposits of immunoglobulins, complement components, and other protein substances in the patient’s tissue by the use of fluorescence-labeled antibodies. DIF is performed when various autoimmune diseases as suspected, such as rheumatoid arthritis, lupus nephritis, autoimmune blistering diseases, and thyroid diseases (15, 16). Although the samples are usually taken from affected lesions, it is possible to detect deposits of autoantibodies and complements in perilesional areas in cases of autoimmune blistering diseases (13). This has the major advantage of maintaining the structure. MMP frequently involves the gingiva and only rarely involves the buccal mucosa. Recently, one study showed the usefulness of DIF using unaffected oral mucosa in MMP (17). However, they did not describe the biopsy regions on the oral mucosa. We selected non-lesional buccal mucosa due to easy access. Biopsying the gingiva is technically more difficult than biopsying the buccal mucosa. In all seven cases, tissue-bound autoantibodies and complements were clearly detected by DIF using non-lesional mucosa (sensitivity: 100%, 95% CI: 59.038–100).

Serological tests are technically easier to perform than histological examinations and DIF, which involve surgical procedures. ELISA has particularly high sensitivity and quickly determines the autoantigens and titers of autoantibodies. Therefore, it can be widely used for most autoimmune diseases. However, the biggest problem with ELISA is the limitation of autoantigens: Recombinant targeting proteins are required for ELISA tests. MMP has multiple autoantigens, and ELISA tests are available for only a few of them. For example, BP180 is one of targeted autoantigen by MMP autoantibodies. However, previous reports demonstrate that 30–42% of MMP autoantibodies are detected by BP180-NC16A ELISA (5, 12, 18). To overcome this problem, Izumi et al. reported the usefulness of recombinant full-length BP180 ELISA (18). The full-length BP180 ELISA detects autoantibodies not only to the NC16A domain but also to parts of BP180 outside of the NC16A domain. The sensitivity is increased from 42% in the BP180-NC16A ELISA into 75% in the full-length BP180 ELISA. Immunoblotting using normal human skin extract or IIF using normal human skin cover greater ranges of antigens. However, these tests are less sensitive than ELISA, and many of the autoantibodies are rarely detected. Indeed, none of the autoantibodies were detected by IIF or immunoblotting in this study (sensitivity: 0%, 95% CI: 0–40.962). Because of these diagnostic difficulties, it frequently takes time to diagnose and start treatments for most MMP patients. Moreover, the diagnosis delay was reported to be site specific. The oral mucosal lesions are thought to be more difficult to detect than the skin lesions such as those on limbs or trunk (19). The average time between initial symptom and diagnosis was more than 2 years in our study.

In conclusion, to confirm the diagnosis of MMP, we highly recommend DIF using non-lesional buccal mucosa. The procedure is technically easy and has high diagnostic value.

## Ethics Statement

All studies conformed to the guidelines of the medical ethics committee of Hokkaido University and the Declaration of Helsinki Principles. Written informed consent was obtained before any samples were collected. A full review and approval by an ethics committee of Hokkaido University were not required, according to local guidelines.

## Author Contributions

MK and JS participated in data generation and analysis, and MK and HI wrote the paper. MK, HI, HU, IU, and YK contributed samples and clinical data. HI, HU, YK, and HS supervised the study. All the authors had final approval of the submission.

## Conflict of Interest Statement

The authors declare that the research was conducted in the absence of any commercial or financial relationships that would pose potential conflicts of interest.

## Abbreviations

MMP, mucous membrane pemphigoid; BMZ, basement membrane zone; IIF, indirect immunofluorescence; DIF, direct immunofluorescence.
